# Supplementary figures and images for: A structural signature motif enlightens the origin and diversification of nuclear receptors
Source: PLoS Genet. 2021 Apr 21;17(4):e1009492. doi: 10.1371/journal.pgen.1009492 (PMC8092661; doi:10.1371/journal.pgen.1009492)

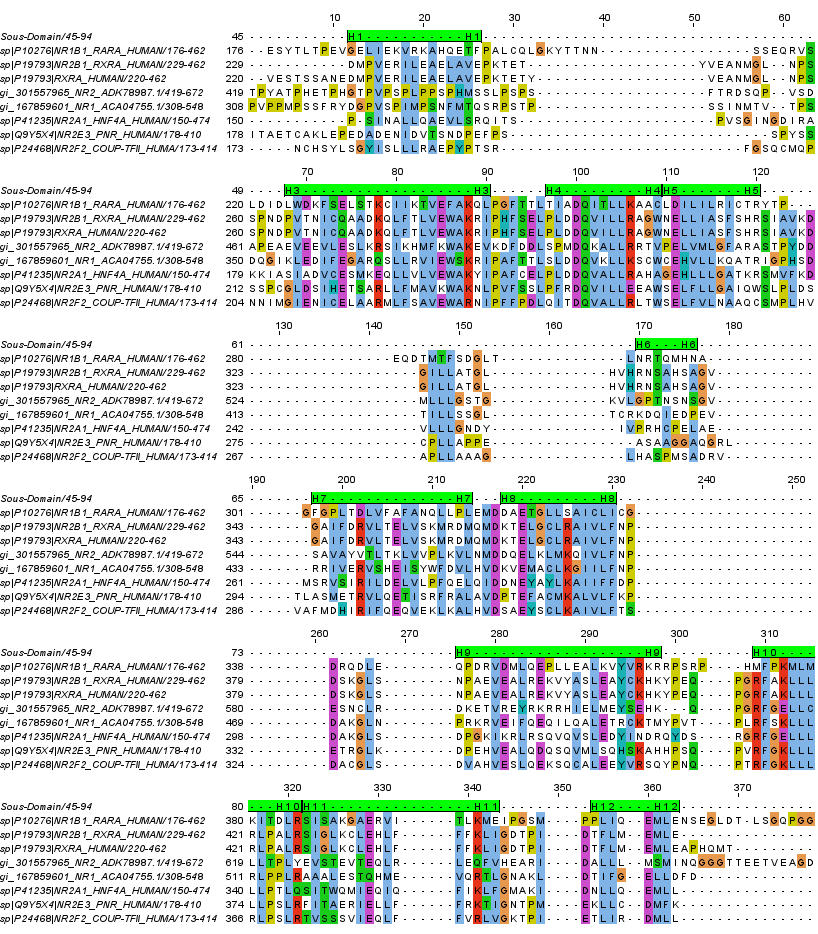

Supplement: S1 Fig — Alignment generated with Clustal Omega software and manually corrected. (TIF) [file pgen.1009492.s001.TIF]

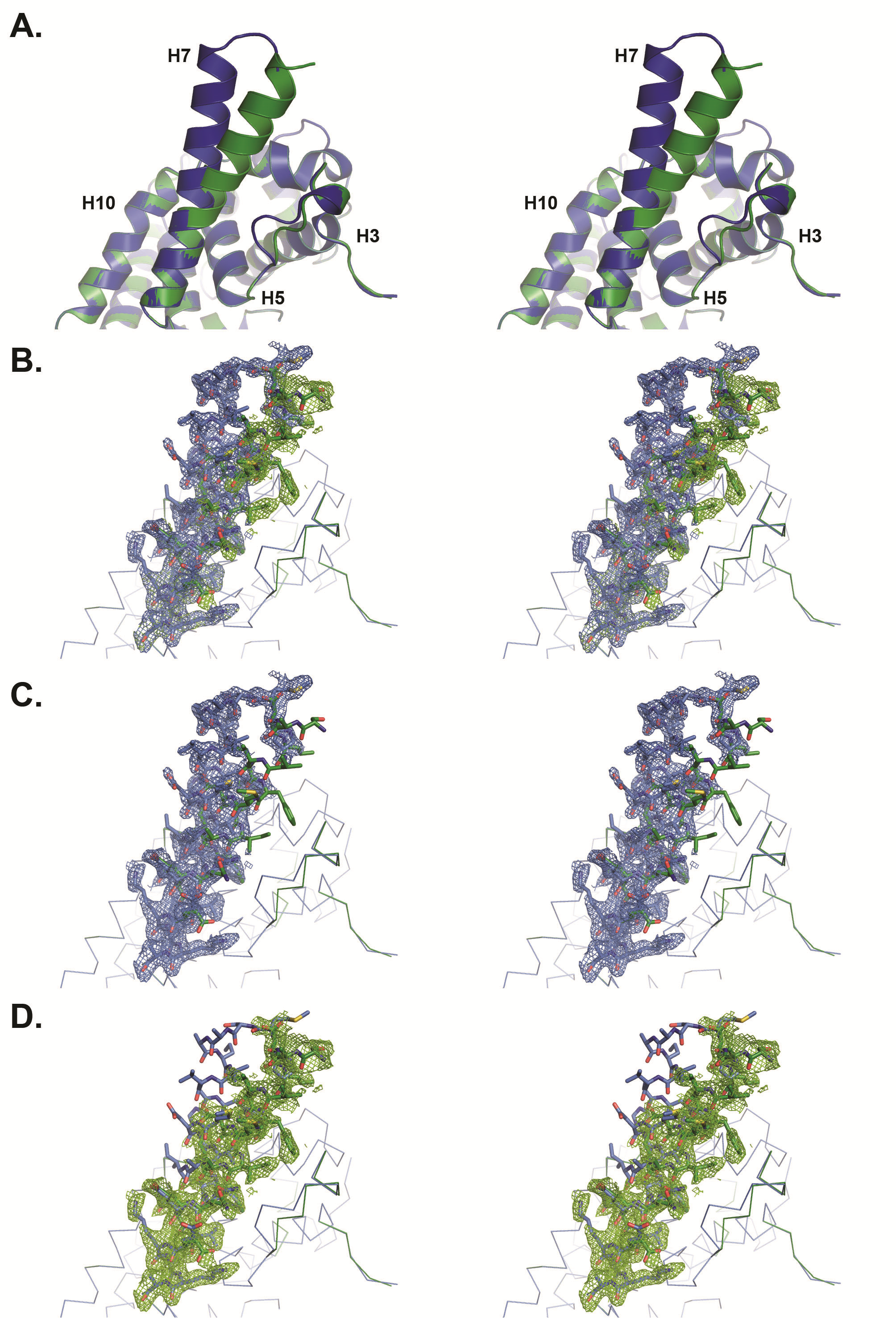

Supplement: S2 Fig — (A) Ribbon diagram showing the double conformation of helix H7. Conformation A is colored in green, and conformation B in blue in all images. All images are presented in cross-eye stereo. (B) 2mFo-DFc electron density map contoured at 0.5 sigma (0.16 e-/Å3) around helix H7. The density for conformation B is shown in blue, and the supplemental density for conformation A is shown in green. (C) 2mFo-DFc electron density map contoured at 0.5 sigma (0.16 e-/Å3) around conformation B only of helix H7. (D) 2mFo-DFc electron density map contoured at 0.5 sigma (0.16 e-/Å3) around conformation A only of helix H7. (TIF) [file pgen.1009492.s002.TIF]

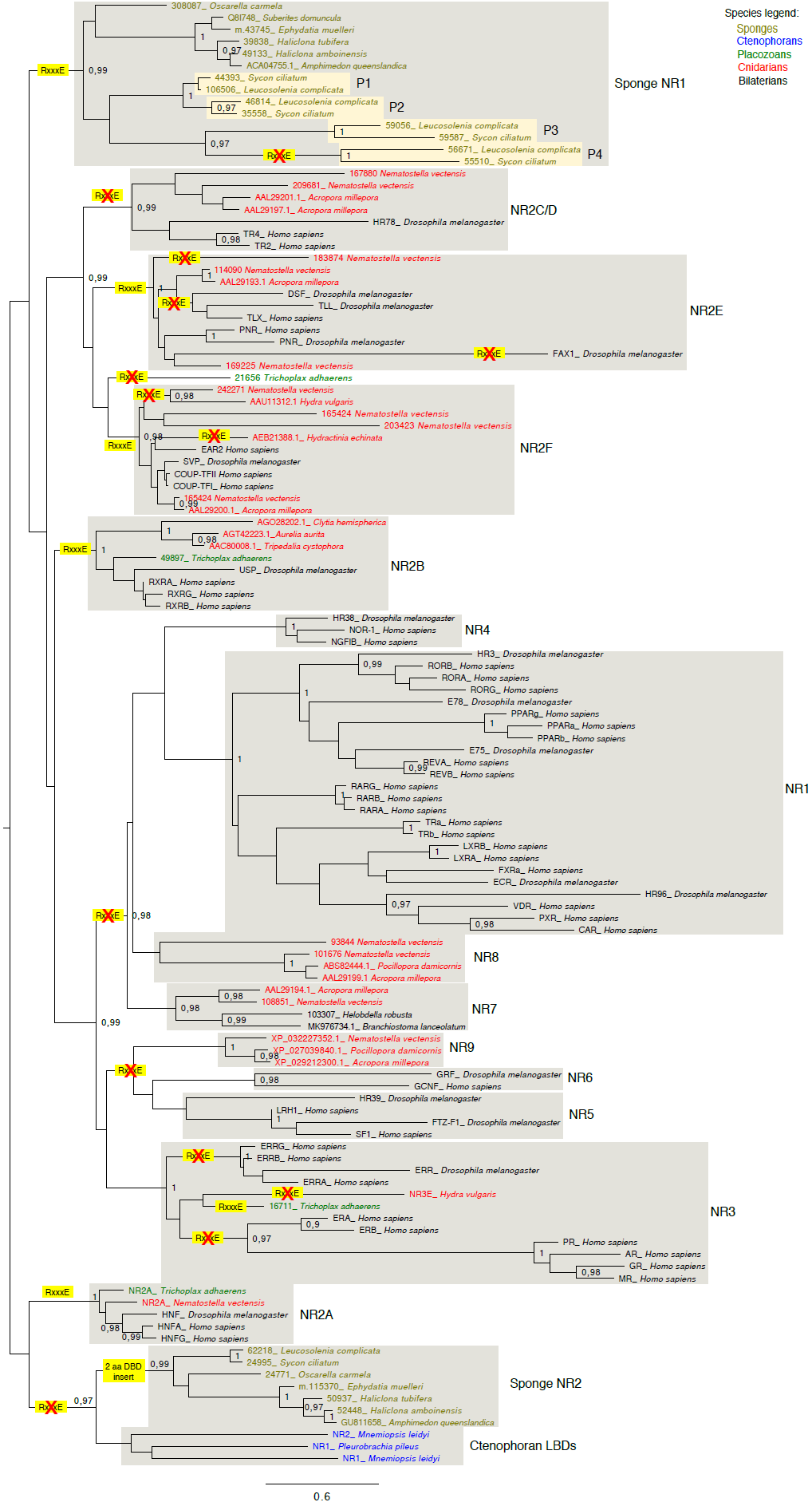

Supplement: S3 Fig — Classical and newly defined NR families are indicated with grey boxes. Species sequences are colored according to the five main metazoan groups they belong to. Branch support values are assessed by approximate likelihood ratio test (aLRT) and are show only for nodes that are considered fully robust (above 0.97). Occurrence of the RxxxE motif is also indicated. Our improved sampling also confirms that there are two cnidarian-specific NR groups. The first one is NR8, that branches at the basis of the crow group containing the bilaterian-specific NR1/NR4 group. The NR1/4/8 group is sister to the eumetazoan NR7 group, which has undergone lineage specific losses in vertebrates and arthropods, and is therefore represented here by sequences from the leech Helobdella robusta and Branchiostoma lanceolatum. Although the internal branching between NR1, N4, NR7 and NR8 are not supported by strong branch support, they are consistent with the last global phylogenetic analysis of the NR family [9]. In this study, the NR7 group was informally called “INRa” and the NR7 group was called “INRb”. Also, there is an additional cnidarian-specific group branching at the basis of the bilaterian NR5 and NR6 groups, which was unformally called “cSF-1”. Here it is named NR9, since it is at the basis of NR5 and NR6, and two additional sequences from the anthozoan cnidarians Pocillopora damicornis and Acropora millepora are provided. Concerning the NR3 family, cnidarian sequences were identified [88] and here, the Hydra sequence was used as a representative of this group. Interestingly, among basal groups, the loss of the RxxxE motif is always associated with other deviations from the ancestral state. In the NR2A group, the RxxxE motif has been lost in a group comprising the Ctenophoran sequences, that completely lost their DBD ((Reitzel et al., 2011); in blue, as well as in all of the sponges sequences, in which the DBD diverges through a 2 to 5 residue insert. Inside the sponge SpNR1 group, it [file pgen.1009492.s003.TIF]

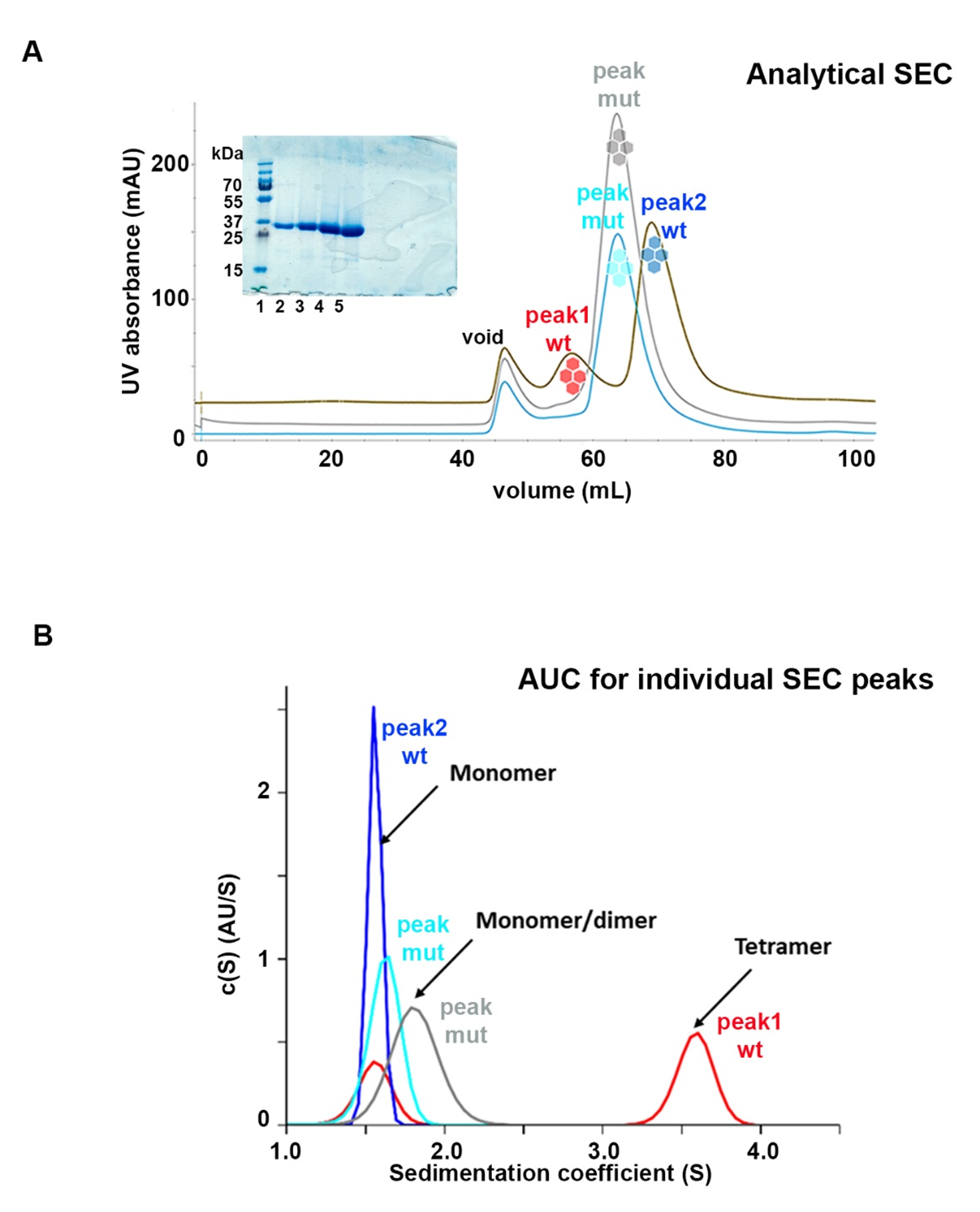

Supplement: S4 Fig — (A) Size-exclusion chromatograms from analytical SEC chromatography on a S200 10/300 Superdex column for wt RXRα LBD (olive line), E352A RXRα LBD (cyan line) and ΔE352 RXRα LBD (grey line). Peaks are named void for the peak roughly corresponding to the exclusion volume of the column, ‘peak1 wt’ and ‘peak2 wt’ for the two peaks of wt RXRα LBD and ‘peak mut’ for the peak of the mutant constructs. The peaks are further marked by symbols in colors that are then consistently used for the panel B; red for peak1 wt, blue for peak2 wt, cyan for E352A RXRα and grey for ΔE352 RXRα. The polyacrylamide SDS gel depicted in the insert indicates the high purity of the samples. (B) Differential sedimentation coefficient distribution c(S) as a function of the sedimentation coefficient S (in Svedberg) obtained from AUC experiments at 1μM, showing the c(S) distribution for peak 1 wt (red curve) and peak 2 wt’ (blue curve) of the wt RXRα LBD SEC peaks, as well as for peak mut of E352A RXRα LBD SEC peak (cyan curve) and ΔE352 RXRα LBD SEC peak (grey curve). (TIF) [file pgen.1009492.s004.TIF]

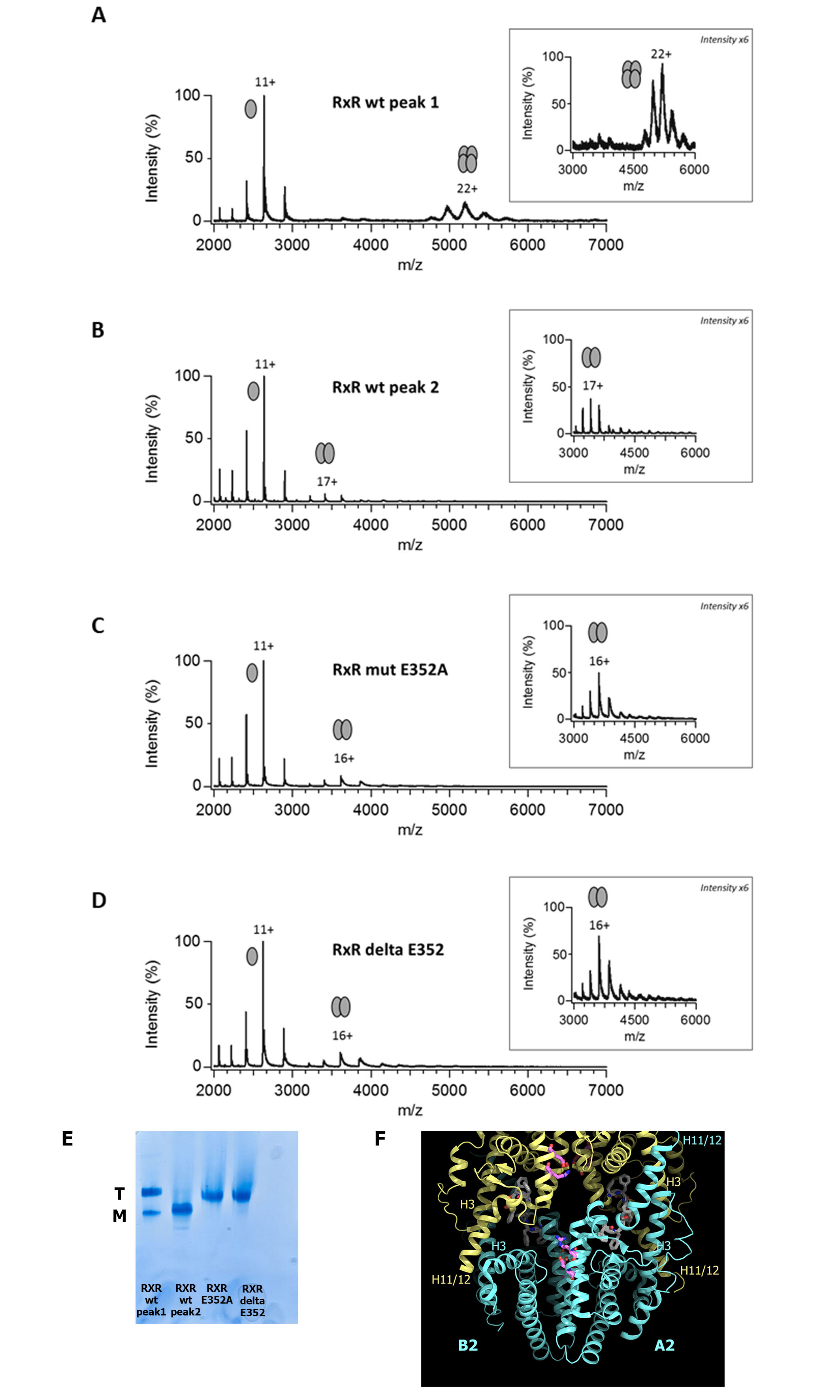

Supplement: S5 Fig — (A-D) Native mass-spectrometric analysis of RXR LBD wt and mutants performed at a voltage 60 V of the SEC peaks. The inserts on the right show an enlarged view (by a factor of 6) of the m/z region that encompasses the dimer m/z peaks centered around the 16+ species. Striking is the complete absence of tetramers for the RXR LBD mutant species. (E) Native polyacrylamide gel electrophoresis of the different SEC peaks of wt RXRα LBD (peak 1 and peak2) and of the mutant E352A RXRα LBD and ΔE352 RXRα LBD. T and M specifies the tetrameric and the monomeric species, respectively. (F) Cartoon representation of tetrameric RXRα as observed in the crystal structure of RXRα LBD (PDBcode 1G1U). The two homodimers of the tetramer (A1/B1 and A2/B2) are shown in yellow and cyan, respectively. The Arg and Glu residues of the π-turn are shown as sticks, colored in magenta for carbon, blue for nitrogen and red for oxygen. The triple Phe residues of H11 are depicted in as sticks colored in grey for carbon. Important helices of the tetramer interface are indicated. (TIF) [file pgen.1009492.s005.TIF]

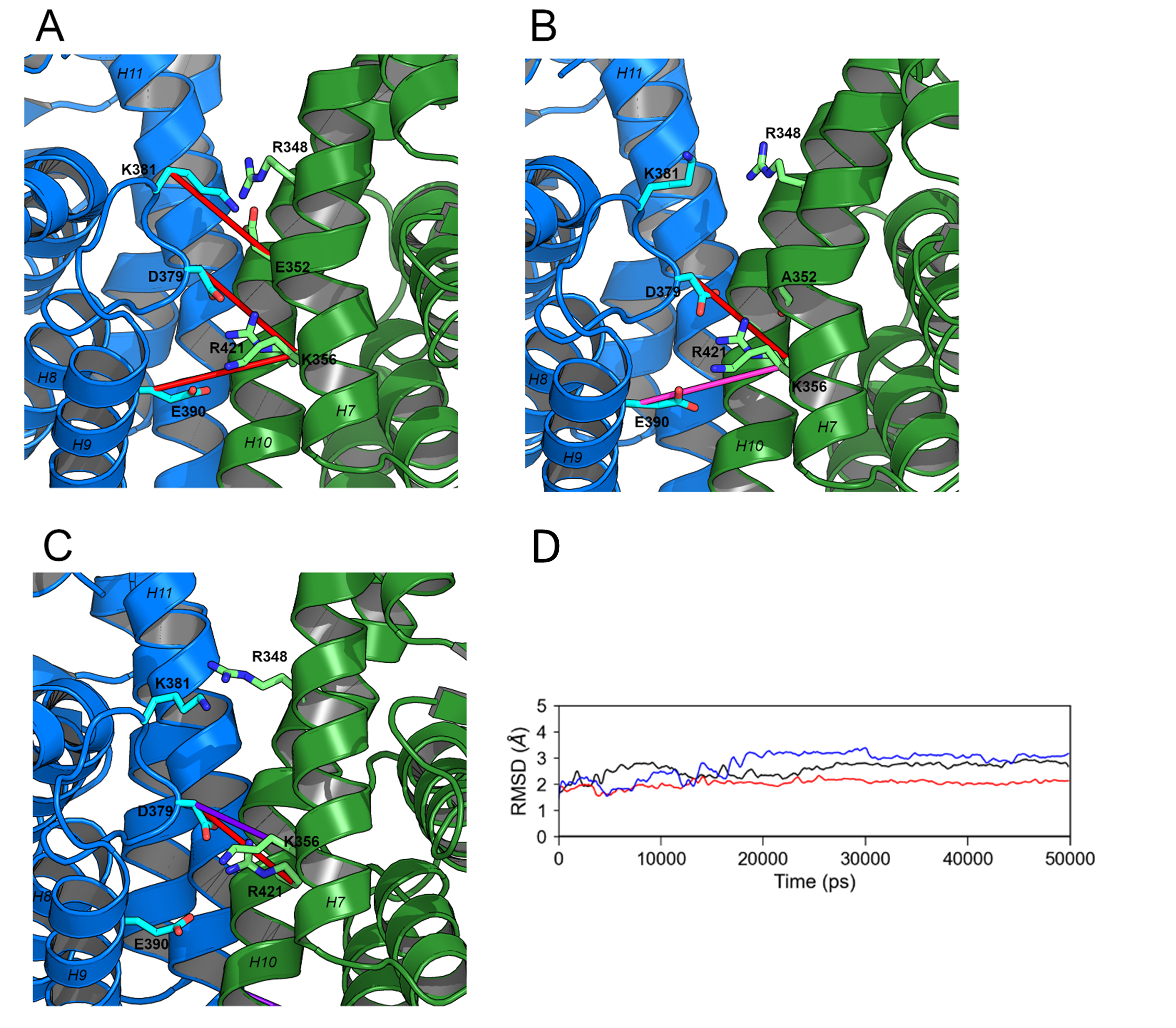

Supplement: S6 Fig — The electrostatics interactions were analyzed during the MD simulations of (A) wt, (B) E352A and (C) ΔE352 RXRa LBD homodimer complex. The percentage of interaction presence is represented with lines connecting the corresponding residues, in red (between 75 and 100% of simulation time), purple (between 50 and 75% of simulation time) and pink (between 25 and 50% of simulation time). (D) Time evolution of the Ca-RMSD of the wt RXRα LBD (black line), E352A RXRα LBD (blue line) and ΔE352 RXRα LBD (red line), represented in black, red and blue respectively, over the course of the 50ns molecular dynamics simulations. (TIF) [file pgen.1009492.s006.TIF]

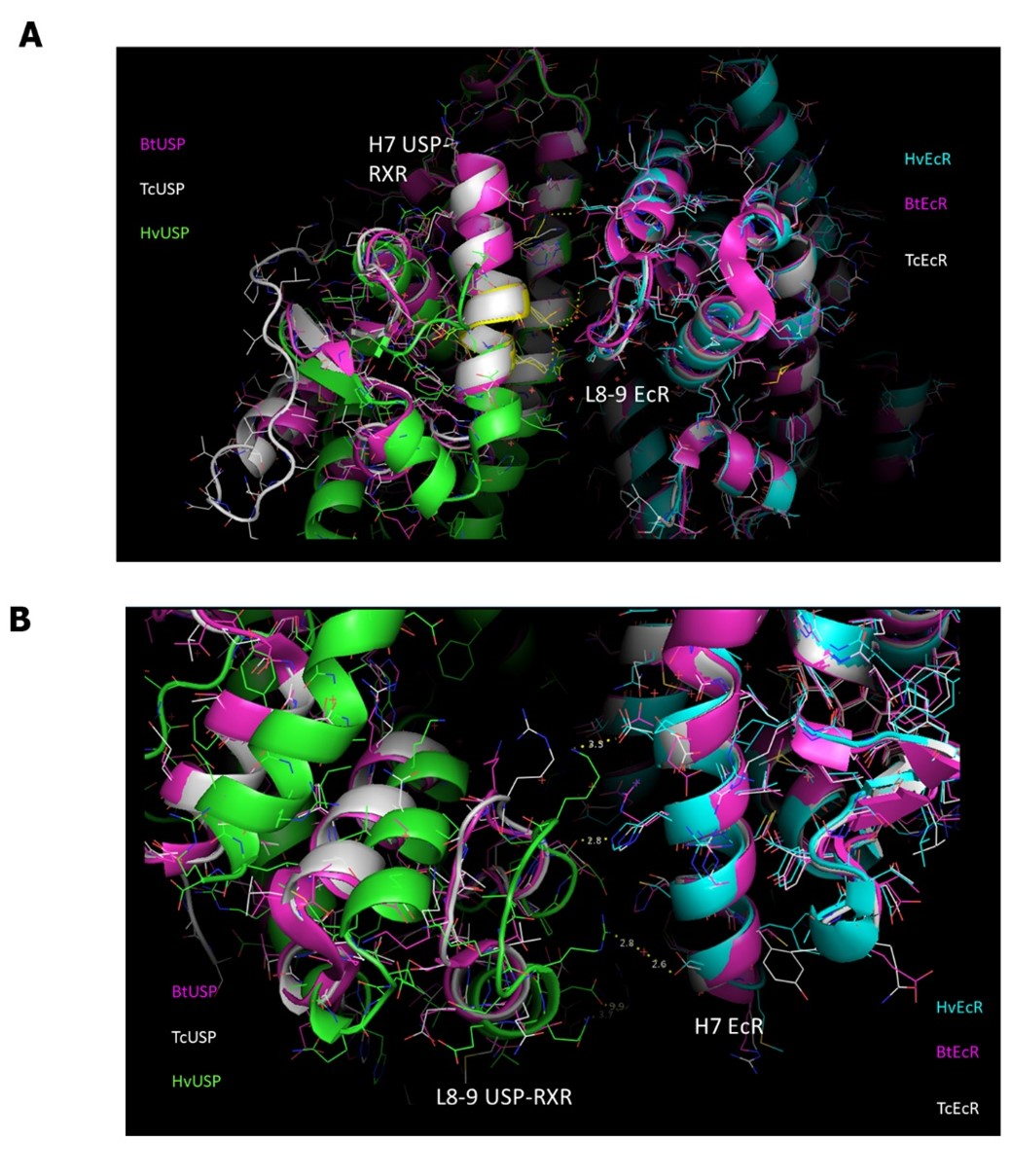

Supplement: S7 Fig — (A-B) Detailed views of heterodimeric interactions between EcR and USP-RXR LBD structures of different insect species, the more recent species Heliothis virescens (Hv), and the more basal species Tribolium castaneum (Tc) and Bemisia tabaci (Bt). The structure of HvEcR/USP-RXR is the one bound to 20-hydroxyecdysone (PDBcode 2R40), that of TcEcR/USP-RXR is bound to ponasterone A (PDBcode 2NXX), that of BtEcR/USP-RXR is bound to ponasterone A (PDBcode 1Z5X). In (A), the view shows the interactions of USP-RXR H7 with the EcR partner loop H8-H9 which are rather conserved among species. In (B), the view shows the large differences in interaction distances between EcR H7 helix and the partner USP-RXR at the level of its loop H8-H9. Intermolecular interactions between EcR H7 and USP-RXR H8-H9 loop can only be formed for HvEcR/USP-RXR and not for TcEcR/USP-RXR and BtEcR/USP-RXR, due to too large distances between these structural elements. (TIF) [file pgen.1009492.s007.TIF]

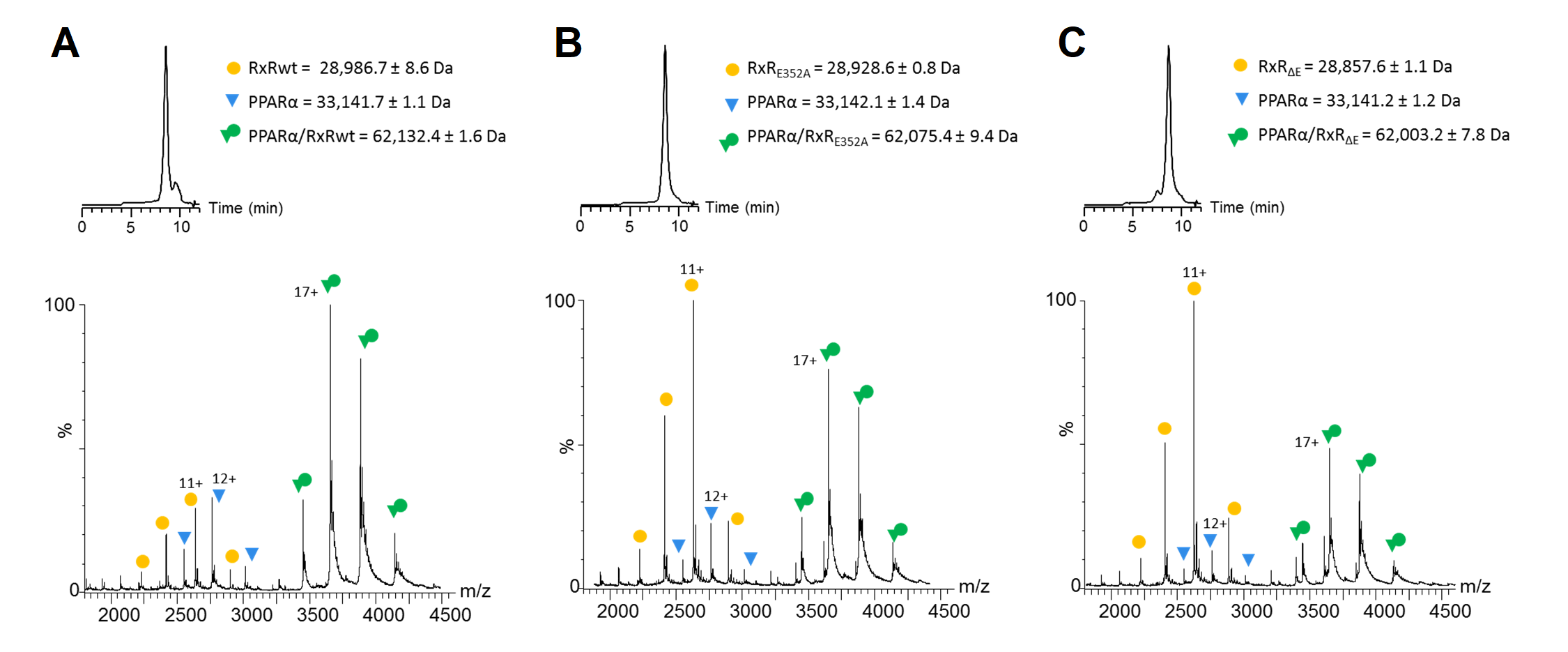

Supplement: S8 Fig — Size-exclusion chromatography (SEC)-coupled native mass spectrometric (MS) analysis for complex mixture of PPARα LBD with either (A) wt RXRα LBD, (B) E352A RXRα LBD or (C) ΔE352 RXRα LBD. The isolated RXRα LBD (wt or mutants) is depicted in yellow, isolated PPARα LBD in blue and heterodimeric PPARα/RXRα LBDs in green. The raw mass spectra are depicted together with the size-exclusion chromatograms of wt RXRα LBD and of mutants RXRα LBD. Integration of the mass spectra is performed over the whole peak of the chromatogram. (TIF) [file pgen.1009492.s008.TIF]
