# Supplementary material for: A structural signature motif enlightens the origin and diversification of nuclear receptors
Source: PLoS Genet. 2021 Apr 21;17(4):e1009492. doi: 10.1371/journal.pgen.1009492 (PMC8092661; doi:10.1371/journal.pgen.1009492)
Supplement: Attachment — Submitted filename: 108363_1_rebuttal_1913144_qjlmbk.pdf [file pgen.1009492.s013.pdf]

We first would like to thank the three referees for the quality of their review and give in the following our revision plan for further submission to eLife, one of the affiliated journals of Review Commons.

**Referee 1**

1. *The pi-helix appears to be important for the function and oligomerization (dimerization) of HNF4 LBD and oligomerization (tetramerization) RXR LBD, respectively. However, other nuclear receptors such as the NR3s (ER, GR, etc.) that form homodimers apparently lack a pi-helix in helix 7 of their LBD. This indicates the pi-helix may be important for some modern nuclear receptor oligomers but not all. The authors should discuss this point.*

Our analyses suggest that the most basal nuclear receptors HNF4 and RXR do possess a $\pi$- turn that represents a critical part of their homodimeric interface. The $\pi$-turn of RXR evolved then as being an essential feature allowing this receptor to heterodimerize with more recent nuclear receptors, such as those belonging to subfamilies NR1 and NR4. The steroid nuclear receptors of subfamily NR3 encompasses the estrogen receptor ER, the estrogen-related receptor ERR and the oxosteroid nuclear receptors (GR, AR, MR and PR). Concerning ER and ERR, they do form homodimer even though they do not possess a $\pi$-turn, nor the RxxxE motif. The homodimerization interface for these two receptors is composed of the same secondary structural elements as seen in RXR and HNF4. However, ER and ERR evolved in a way that they underwent compensatory mutations leading to the disappearance of the RxxxE motif. However most other residues at the dimerization interface remained conserved and allow numerous complementary electrostatic and van der Waals interactions. On the other hand, the later evolved oxosteroid nuclear receptors are different in their dimerization properties. Their ligand binding domain does not dimerize in the same manner as ER and ERR. In fact, there is a marked sequence difference of the residues at the interface compared to the whole nuclear receptor family and the presence of an additional conserved region at the C-terminal end of the LBD (F-domain) that hampers the oxosteroid receptors to dimerize in a classical way (Williams SP, Sigler PB (1998). Nature and Billas I, Moras D (2013) J Molecular Biology). In our manuscript, we do not relate homodimerization to the presence of a $\pi$-turn, but show that it is central to the dimerization interface of the two most ancient receptors, HNF4 and RXR, and demonstrate that it is essential in the evolution of heterodimerization.

In order to better clarify the important point that the $\pi$-turn is not necessary for the homodimerization of all nuclear receptors, but only for RXR and HNF4, we will emphasize this aspect in the main text, in the discussion part p. 18, as:

*It is important however to emphasize that the $\pi$-turn is not necessary for the homodimerization of all nuclear receptors, but only for RXR and HNF4. In fact, steroid NRs, such as the estrogen receptor (ER) and the estrogen-related receptor (ERR) homodimerize in the absence of $\pi$-turn and RxxxE motif, making use of the same secondary structural elements for building the dimerization interface as RXR and HNF4. From an evolutionary point of view, ER and ERR evolved in a way such as they underwent compensatory mutations leading to the disappearance of the RxxxE motif, but conserving most of the other interacting residues. On the other hand, the later evolved oxosteroid nuclear receptors (AR, GR, MR and PR) are different in their dimerization properties. Their ligand binding domain does not dimerize in the same manner as ER and ERR. In fact, there is a marked sequence difference of the residues at the interface compared to the whole nuclear receptor family and the presence of an additional conserved region at the C-terminal end of the LBD that hampers the oxosteroid receptors to dimerize in a classical way that still needs to be uncovered.*

2. *Please check the literature to ensure the monomeric/dimeric state in Fig. 4 is accurate. For example, to this reviewer's knowledge the RORs are thought to function as monomers but the authors indicate they are homodimers. Similarly, NR4A3 (NOR-1) is listed to function as a heterodimer (with RXR) but published work indicates it does not interact with RXR.*

We re-checked the literature using the IUPHAR/BPS guide to pharmacology (Alexander et al., 2019) completed by manual literature searches. Concerning ROR, it functions indeed like a monomer (Laudet & Gronemeyer, The Nuclear Receptor Factsbook, Academic Press 2002) Concerning NOR-1: There are indeed no experimental evidence that NOR-1 heterodimerizes with RXR. However, NOR-1 belongs to the same subfamily as NGFI-B and Nurr1 which were shown to heterodimerize with RXR (Zetterstroem RH et al, Mol Endo 1996). NOR-1 might be an exception to this subfamily. To take into account the reviewer's comment, we have performed an alternative mapping with ROR and NOR-1 (NR4A3) encoded as monomer. As you can see in the comparison below, this does not change significantly the character estimation at deep ancestral nodes. To take into account the reviewer's comments we have modified Figure 4 and replaced the character mapping by this new one. See below the new version (right panel, now Fig 3B) compared to the old Figure 4 (left panel).

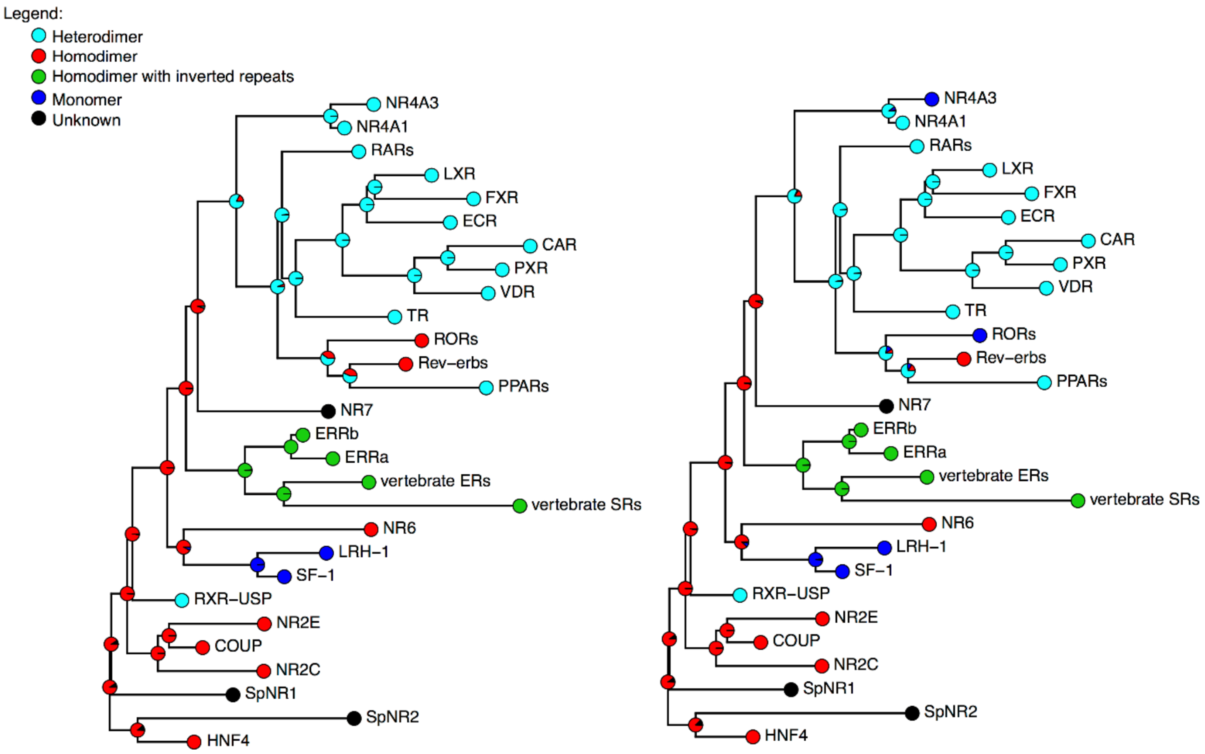


*3. RXR LBD alone exists as a mixture of monomer, homodimer and homotetramer. Mutagenesis of RXR pi-helix caused the appearance of a single AUC species that is attributed to a monomer/dimer. Which protein interaction interface remains intact? There are two ways RXR could dimerize in the pi-helix mutant. The authors speculate the tetramerization surface is negatively impacted by the pi-helix mutant (and the helix 7/11 dimer interface remains intact) but have not ruled out that the helix 7/11 dimer surface is negatively impacted (and the disulfide linked tetramer interface links two RXR*

*molecules remains intact). One way to assess this would be to perform studies in the presence of a reducing agent to determine if the disulfide bond that connects the tetramer interface-which prior work has shown is broken upon addition of reducing agent-regulates the solution properties of the pi-helix mutant. If dimerization occurs through helix 7/11, addition of a reducing agent should have no effect on the AUC, SEC, or native MS data. This is an important point, because in the abstract the authors claim the pi-helix regulates RXR homodimerization; however, the biochemical data presented by the authors indicate it regulates homotetramerization (and perhaps not through the canonical dimer interface).*

RXR LBD is effectively known to exist in the form of a monomer, a homodimer and a tetramer. Our AUC experiments show that for wild-type RXR, the protein is in the form of monomers or of tetramers, the latter being dimer of homodimers. When mutating RXR, the homodimer is destabilized and the tetramer even more. The fact that we do see the appearance of a monomer/dimer species is linked to the disappearance of tetrameric species.

The mutation of the π-turn does not prevent homodimer formation since the interface also encompasses other mostly conserved and hydrophobic residues (Wurtz J-M et al (1996). Nature Structural Biology). As it was suggested by the MD studies the helix 7/11 dimer interface remains intact, but is severely weakened. This is in agreement with our observations that suggest that the dimer of dimer (the tetramer) is destabilized. Indeed, to form a tetramer, the RXR homodimer needs to be nicely stable. Note that there are no disulfide bonds that hold together the RXR molecules in the tetramer and the tetramer interface can be easily destabilized. This is attested by classical mass spectrometry analysis in denaturing conditions (water/acetonitrile +1% formic acid) where RXR is detected as a monomer, while RXR is a tetramer in native conditions (ammonium acetate) which demonstrates the noncovalent nature of the heterodimer. In addition, the structural analyses of the crystal structure of RXR tetramer does not indicate any covalent bonds holding together the different subunits in the tetramer. Further, our measurements are performed under reducing conditions provided by the addition of the reducing agent TCEP at all steps of experiments, and thus the experiments suggested are already addressed in this respect. Finally, our mass spectrometric experiments of RXR performed under denaturing conditions indicate that RXR is in the form of a monomer.

We changed on page 14 to "*However, residues of the π-turn interact with helix H11 and help stabilizing its conformation. The mutation of the conserved Glu residue of the π-turn does not prevent homodimer formation since the interface also encompasses other mostly conserved and hydrophobic residues. However, it is likely to destabilize helix H11 and as a consequence to weaken the homodimer interface, enough to lead to the destabilization of the tetramerization interface, as observed experimentally*".

*4. The authors claim that the pi-helix enables RXR to heterodimerize with other nuclear receptors, but there is no biochemical data to support this. It should be straightforward for the authors to test this using the same biochemical methods reported for RXR.*

We thank Referee 1 for the suggestion. Additional experiments focused on a heterodimer indeed provide an added value to the manuscript by providing solid experimental evidence of the role of the π-turn motif in RXR for heterodimerization. We have chosen to study the heterodimerization of RXR wild-type and mutant (E352A and deltaE) LBDs with PPARa LBD, using size-exclusion chromatograph (SEC)-coupled to native mass-spectrometry techniques. This is the technique of choice for this

demonstration, since it is sensitive, precise and allows a relative quantification of the heterodimer populations in the complexes involving RXRα (wt or mutants) and its partner PPARα. We indeed do not expect a complete disappearance of the heterodimer population between mutant RXR and PPARα, but a reduction in their relative population compared to the heterodimer with wt RXRα. SEC-native MS analyses have been performed to quantify the relative heterodimer populations as a function of the RXRα constructs. These new data are integrated to the revised version of the manuscript in Figure 6 and in Suppl. Fig. 8, in the result part as well as in the discussion part on page 19.

5. To ensure the computational analysis that was performed is rigorous, reproducible, and indeed representative of the entire nuclear receptor superfamily (a main emphasis of this manuscript), I recommend the following:

Our computational analysis is indeed rigorous and reproducible and to assess this we will provide the elements as described below in points 5A and 5C.

5A. Sequences and alignments used to general phylogenetic trees should be made available as supporting datasets.

5A. Sequences and alignments will be made available either on a Dryad repository, or directly as supplemental information related to the concerned figure. As indicated in the Dryad help page (https://datadryad.org/docs/JournalLookup.pdf), we will wait until MS submission and follow eLife's instruction about data submission if our manuscript is finally published by this journal.

5B. *It is not clear how the measurement of the presence of absence of a pi-helix was determined (visually for representative structures or in an unbiased approach using a computer program?) and whether the observations are representative of all structures for each nuclear receptor discussed (e.g., do all RXR crystal structures show a pi-helix or just a certain percentage?). It should be relatively straightforward for the authors to use a program such as DSSP in an automated manner to assess the secondary structure content of all nuclear receptor crystal structures and provide statistical measurement (e.g., a pi-helix was found in RXR crystal structures X% of the time).*

The $\pi$-turn was determined from inspection of all available crystal structures of nuclear receptors that contain a RXXXE motif, without any exception (105 structures in the PDB database: 6 HNF4-alpha LBD, 1 HNF4-gamma LBD, 80 RXR-alpha LBD, 6 RXR-beta LBD, 1 RXR-gamma LBD, 11 USP LBD), as the presence of a $\pi$-turn is intimately linked to the presence of this motif. We have found a $\pi$-turn in all RXR and HNF4 crystal structures, but none in COUP-TF and PNR structures. DSSP does not provide information on presence or absence of $\pi$-turn in nuclear receptors, the reason being that this element is too short and only represents a single $\pi$-type hydrogen bonds, i.e. one turn of a $\pi$-helix (the minimal length of a $\pi$-helix is typically seven residues as described in Ludwiczak et al, Sci. Reports, 2019).

5C. *Any scripts or code used to perform the computational analyses should also be made available as supporting information.*

R scripts used for character mapping on Fig. 3 is now available as supplementary information. Regarding the phylogenetic analysis and molecular dynamics simulations, all parameters from the used open source programs are indicated, making the analysis reproducible.

*6. I do not believe that homology modeling of SpNR1 is appropriate to determine whether or not it contains a subtle structural feature such as a pi-helix segment. The methods state that crystal structures either containing or lacking a pi-helix were used as modeling templates, which produced SpNR1 models with or without a pi-helix, respectively, which is expected. It is not sufficient to say that the pi-helix-containing model displayed the lowest predicted energy model and was therefore cited as support that SpNR1 contains a pi-helix. The only way to determine this would be to solve a crystal structure of SpNR1*

We are aware of the limitations of homology modeling, especially in predicting fine structural features such as π-turn. Nevertheless, homology modeling of SpNR1 was attempted to have a neutral analysis in the absence of crystal structure of SpNR1. In fact, we tried, but unsuccessfully, to crystallize this receptor LBD. The SpNR1 LBD structure would have brought the experimental confirmation. We address the issue of homology modeling in more details in the reply to Referee 3, point 5 and in the manuscript accordingly.

**Replies to the Minor comments of Referee 1:**

1 *.Page 5: the authors state the LBD fold is "unique", but "conserved" might be more appropriate.*

OK, done

2.*Throughout the manuscript "pi-helix", "pi-bulge", and "pi-turn" are used to refer to the same structural element. Also 3(10) helix and 3(10) turn are used interchangeably. I recommend changing all to "pi-helix" and "3(10) helix" because this is the nomenclature detected by DSSP*.

3(10) helix will be used throughout the manuscript.

Concerning π-turn or π-helix, we will keep the terminology π-turn throughout the manuscript, because this structural feature represents only a short segment of a π-helix. As specified in Ludwiczak et al, Sci. Reports, 2019, a π-helix has a minimal length of seven residues (two consecutive π-type hydrogen bonds) and can be up to 14 residues long in rare cases. In our case, for nuclear receptor, we have a short five residue long a π-type segment that is typically named α/π-bulge or π-turn. This nomenclature better describe our observation. Accordingly, we have modified the first paragraph of the Results part to better define the concept of π-helix and π-turn.

3. *In the figures, it appears that some sort of sequence alignment numbering is used when calling out specific residues, which makes it difficult to relate the numbering to specific nuclear receptor crystal structures. I recommend using the actual amino acid sequence numbering that is specific to each nuclear receptor instead of the position in the sequence alignment*.

We have carefully considered this suggestion but we feel this would be prone to less rather than more clarity, forcing us to name the same position with different numbers which will confuse the reader. We do emphasize that we have included a Table (Table 1) that provides the equivalence in numbering in each NR for all positions discussed in this paper.

4. *Fig 2: Panel A the Turn 3(10) label is clipped. Panel D is missing an arrow for the Turn 3(10).*

This is done.

5.*The phrase that the pi-helix "will be favored only when they are associated with a functional advantage" is a hypothesis put forward by the cited articles and should therefore not be stated as a fact.*

We have modified the sentence to: "*is considered to be favored only when they are associated with a functional advantage*" to show that this remain speculative (although very likely).

6.*Ensure that supporting information figures are provided to clearly justify any sequence-specific statements in the results. For example, to justify this: "The analysis of sponge nuclear receptor sequences show the presence of the RxxxE motif in helix H7 of SpNR1, but not of SpNR2."*

We have provided in a revised version of Supp Fig S1 a sequence alignment that allows to substantiate this conclusion.

7.*Page 7-8 discusses "markers of dimerization" for class I and II nuclear receptors corroborate some observations, but the specific "markers" are not detailed.*

These markers are discussed in full in Brelivet et al., 2004. We now list them in parentheses in this sentence, as requested.

On page 8, we have added "…(E5, W40, K/R55, R/K93, R105 for class I NRs; E/D42, R62, H/R/K90 for class II NRs and E50 and R105 universally conserved)."

8.*Fig 3 gives information in absolutes: pi-helix is present or absent. Can a probability be defined rather than an absolute present or absent? Also, this figure is very complex and hard to understand; the colors and symbols used should be shown in a graphical legend within the figure instead of descriptions in the figure legend text.*

We have simplified the Figure 3 using the same tree topology as in figure 4 and using probabilistic mapping for the presence/absence of the RxxxE motif. See the updated figure 3 below and answer to comment 9.

9.*Fig 4: It is not clear how this information connects to the pi-helix emphasis of this manuscript. Only 2 nuclear receptor subfamilies show a pi-helix, but there are many other nuclear receptors that form homodimers or heterodimers? I also recommend using a graphical legend (instead of descriptions in the figure legend text) to describe each color corresponds to in the figure.*

The figure was intended to explore the connection between presence/absence of a $\pi$-turn and the various dimerization modes encountered throughout the family, but we recognize it contained too many information and was not very clear. We agree that the comparison would be easier with the same tree topology and therefore merged the updated figure 3 with the updated figure 4 (now panels A and B respectively in the new Figure 3). As the probabilistic mapping clearly favor the ancestry of the RxxxE motif and the $\pi$-turn we present now only this hypothesis which is now unambiguously supported. We thank the reviewer for this useful suggestion that allowed us to streamline the manuscript on that level. As requested, we now included a graphical legend to the figure.

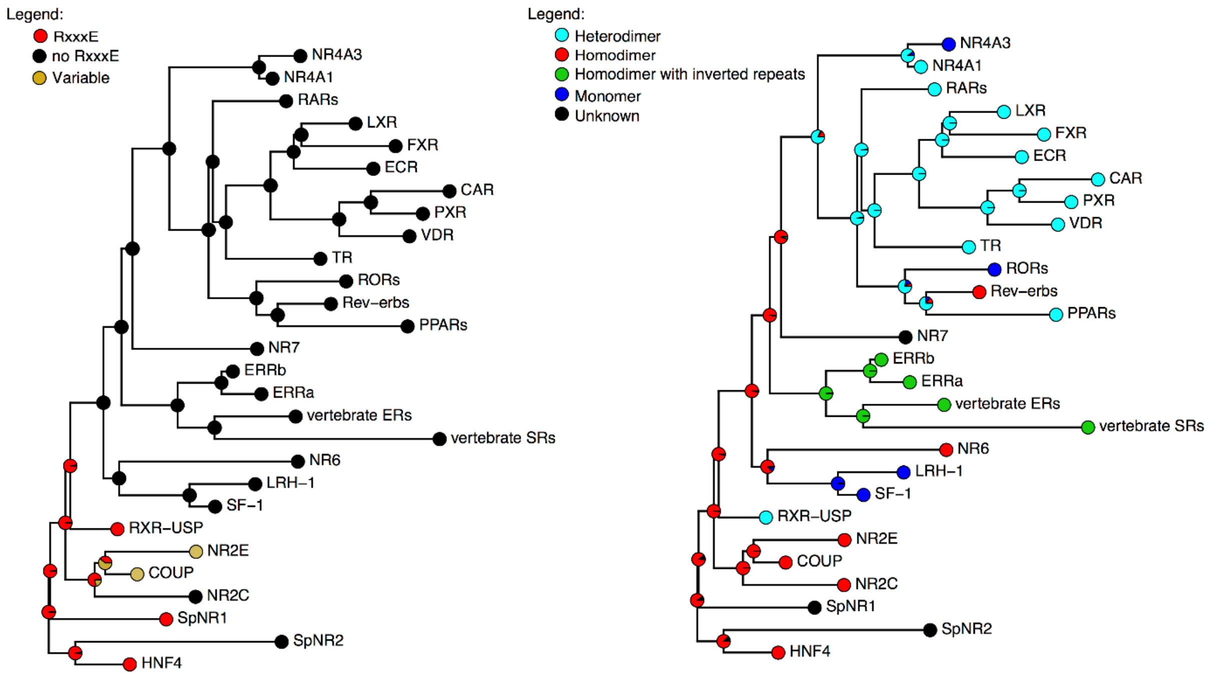

**Figure 3: Evolution of the RxxxE motif compared to the dimerization modes during the history of the NR family. A.** Evolution of the RxxxE motif, as reconstructed using ancestral mapping. **B.** Evolution of dimerization modes. Source files and script are provided in dryad under the following doi: to be added after submission acceptance. The full NR tree with exhaustive mapping of the RxxxE motif is available as Figure S3.

*10.Fig 5: the authors show various contacts in nuclear receptor dimer structures as evidence for homodimerization vs. heterodimerization. Is there any experimental evidence to back this up, such as measured dimerization affinities? Also, it is not clear how the contacts (lines linking specific residues) were measured; if it was visually, this is not a rigorous method and I would recommend using a program like the PISA webserver (interfaces mode > details) as a method that is reproducible. Also, PDB codes should be provided for the structures that were analyzed, and whether the interactions are representative of all dimer structures available for each complex.*

The connections are established using PyMOL (DeLano, W. L. (2002). Pymol: An open-source molecular graphics tool. CCP4 Newsletter On Protein Crystallography, 40, 82-92). The interactions shown are present in the majority of structures, but not necessary in all (more of 75%). We have checked the interactions with PISA for a detailed interaction pattern of the corresponding homo- and heterodimers. We have found that the main interactions represented in the figure are corroborated by the PISA analysis. Additional hydrophobic long-distance

interactions are found using PISA, but they are not essential to our analysis. Therefore, we suggest to keep the figure as it is, since it gives the main electrostatic and H-bond interactions for the various dimers.

11.*The word "peculiar" is used several times to describe things where the use of "specific" may instead be more appropriate*.

Done on p. 11

On p. 18 peculiar removed 2 times

*12.Fig 6: I recommend using consistent colors for all panels; the coloring in A is different that the others. Also, it appears that some samples are called "monomer" in the AUC data but the native MS data indicate they form dimers (blue lines)?*

We improved the figure in order for the main message to be more understandable. For this reason, in the revised version of the manuscript, we have kept only the SEC-MS results in a main figure (renamed Figure 5) and moved the panels A and C to the Suppl. Fig. 4. Please refer to the comments to Referee 3, point 6.

**Referee 2**

***Major comments:***

*Beinsteiner et al. provide extensive sequence analyses, structural analyses and experimental studies to support their hypothesis that the evolution of the ancestral pi-turn motif is important regulatory mechanism in nuclear receptors. These structural analyses [database searches, PDB structure analysis, molecular modeling, molecular dynamics) and experimental studies (mutagenesis followed by oligomer analysis [analytical size-exclusion chromatography, analytical ultracentrifugation, electrospray ionization mass spectrometry] are well done as would be expected from these experience authors. Their detailed analysis of the LBDs dimerization interface through mutation studies of key RXR residues highlights interactions controlling homo- versus heterodimerization.*

*The proposal that the evolution of heterodimers is an exaptation [Gould and Vrba] provides a neat example of this novel evolutionary mechanism.*

*The data and the methods are presented in such a way that they can be reproduced. The experiments adequately replicated and statistical analysis are adequate. Additional experiments are not needed.*

We do thank Referee 2 for this positive appreciation of our manuscript.

***Minor comments:***

*Discussion section line 3. Base instead of basis*

Done

*I think that the authors do not adequately (strongly enough) describe the importance of their research in the Introduction. The key advance as I understand it, is identifying a functional role for the pi-turn in*

*oligomerization of nuclear receptors. This is a new function for the pi-turn motif and they have provided a major (and very interesting) analysis of its evolution. A strong sentence in the Introduction and at the end of the Abstract would alert the reader to the significance of their discovery.*

We have modified the Abstract and the end of the introduction as suggested. In the abstract: "*Altogether, our work allows to identify a functional role for the π-turn in oligomerization of nuclear receptors and reveals how this motif is linked to the emergence of a critical biological function. We conclude that the π-turn can be viewed as a structural exaptation that has contributed to enlarging the functional repertoire of nuclear receptors*."

In the introduction: "*To deepen our understanding of how changes in NR dimerization properties contributed to the diversification of the NR superfamily, we carried out an evolutionary analysis of NR genes, focusing on the evolution of dimerization across the entire NR superfamily. We show here that homodimeric binding was ancestral, whereas heterodimeric and monomeric behaviors evolved later. We further identified a specific structural feature present in helix H7, a so-called π-turn or α/π-bulge, present in RXR and HNF4, as being an ancestral motif critical for the homodimerization of the most ancient NRs. We traced back the complex evolutionary history of this π-turn showing that it was instrumental in the origin of heterodimerization, by allowing a flexible dimerization surface of RXR to accommodate numerous partners with multiple interfaces. The π-turn was originally used for homodimerization, but later was utilized for a different function, namely heterodimerization. This can be considered as a structural exaptation which can be seen as instrumental for the expansion of the repertoire of NR functions*."

**Referee 3**

1.*The authors use several abbreviations without explaining them in the text, making the manuscript hard to read. For instance, the NRs described in the manuscript are referred to by their abbreviations (RAR, TR, PPAR, LXR, RXR, and HNF4), but are not explained in the text.*

The full names of the nuclear receptors and the corresponding abbreviations are given once in the text as they are first mentioned (in particular in the introduction and in the first section of the results).

2.*The classification of NRs described in the 'Introduction' section is hard to follow. For instance, the following line on page 3: "This has allowed to position the root of the NR tree within subfamily II, which is therefore not monophyletic". What is superfamily II, and what does it include?*

We have now indicated that the phylogeny of NRs has allowed to define 7 subfamilies and provided the corresponding references. We have also listed the main receptors present in subfamily II.

Page 3, 3rd paragraph: "*Given their importance and also because their long conserved domains are favorable for phylogenetic analysis, the origin of the NR superfamily have been scrutinized for a long time allowing to define 7 distinct subfamilies (Amero et al., 1992; Escriva et al., 1997; Laudet et al., 1992; Bertrand et al., 2004*)".

Page 3 below: "*This has allowed to position the root of the NR tree within subfamily II that in particular contains the retinoid X receptor (RXR), the hepatocyte nuclear factor 4 (HNF4) and the COUP Transcription Factor 1 (COUP-TF)) and therefore that cannot be considered as monophyletic*."

3.*What is the level of sequence similarity between different NRs (for instance, between RXR and HNF4)? It would be useful to discuss this in the 'Introduction' section.*

The level of similarity between the various NRs is extremely variable between the various domains. Typically distant nuclear receptors exhibit ca. 60% sequence identify in their DNA binding domain and 30% in their ligand binding domain. For the ligand-binding domains of human RXRa and human HNF4-a the sequence identity is 36 % and the similarity 53 %, based on an alignment length of 236 amino acid residues.

We added in paragaph 2 of the introduction the following sentence: "Typically, distant NRs exhibit ca. 60% sequence identity in their DBD and 30% in their LBD. »

4.*Different positions within the sequences of various NRs are referred to multiple times in the text, but their visualization is not easy in Table 1. The authors should consider including a multiple sequence alignment of the discussed NRs as a main figure.*

This multiple alignment is present in the Supplementary Figure S1. The alignments are no longer published in main sections of the papers but referred explicitly and the reader can use them together with the Table of residue numbering to facilitate the reading of the paper.

5. *In this study, homology modeling is employed for inferring the presence of π-turns in NRs of unknown structure. However, homology modeling approaches are particularly bad at modeling fine structural features such as π-turns. Do other structure prediction methods such as Robetta and I-TASSER also predict the presence of π-turns in these proteins? Additionally, sequence-based methods (e.g., PiPred) could also be used to substantiate the presence of π-turns.*

We are aware of the limitations of homology modeling and do agree with referee 3. We have tried I-Tasser and Robetta on the NR1 sequence and the best models predicted according to both servers is indeed a model that includes a π-turn in the same region as our homology model, supporting our observations.

Other methods based on sequence such as PiPred do not seem to detect a π-turn in the NR1 structure prediction. This means that the prediction of a π-turn based on the mere sequence is too delicate in nuclear receptors, because i) it is only a short segment, one turn of a π-turn and ii) the presence of a π-turn in the structure strongly depends on the local environment. As for PiPred, we thank the referee for this interesting suggestion, as indicated in the method's publication (Ludwiczak, J., Winski, A., da Silva Neto, A.M. *et al. Sci Rep* 2019) we also tried the server provided https://toolkit.tuebingen.mpg.de/#/tools/quick2d, which seems to be a bundle of different structure prediction methods, however the PiPred method specifically is not present among the algorithms outputted.

6.Figure 6: *The single peak observed in the chromatogram is located between the wild-type monomer and dimer peaks. And MS analysis shows that all proteins have mixtures of dimers and monomers. Could the peak's intermediate position be because of fast interconversion (relative to the chromatographic flow) between monomers and dimers?*

**2020-09-26 Clarification added by Referee 3** *"Apologies for not having been clear. Question nr. 6 refers to Figure 6, panel A and the last paragraph on page 12. The peaks corresponding to the mutants are located between the peaks observed for the wild type. Is this intermediate position possibly because the monomer/dimer conversion is faster than the chromatography in the mutants' case?"*

In the submitted version of Figure 6, panel A, three peaks are observed in the chromatogram of the SEC of wt RXRa, one around 45 mL, one around 60 mL and on later around 70 mL. The peak around 45 mL corresponds to aggregates, the second around 60 mL to the tetrameric species, shown in panels B and C as the red curve and the third one around 70 mL to the monomeric species, shown in panels B and C as the dark blue curve. For the two mutants RXR, the chromatograms in panel A (cyan and grey curves) contain one peak at the void volume (corresponding to aggregates) and another one around 65 mL that is intermediate between the two SEC peaks of wtRXR related to the tetramer and the monomer. These peaks were further characterized using AUC (panel B) and MS (panel C) and are shown to correspond to a mixture of monomers and dimers.

We have clarified this by showing colored symbols below the peaks, red for peak 1, blue for peak 2 of wtRXR and cyan and grey for the 2 mutants. The colors are then consistently kept for all panels.

However, in the revised version of the manuscript, we have kept only the SEC-MS results in a main figure (renamed Figure 5) and moved the panels A and C to the Suppl. Figure 4

7.*Page 18: "Some earlier data suggested that these ancestral receptors play a role as sensors of fatty acid molecules, but no in vivo data have been reported yet to confirm these....". They are not ancestral as they are still found in extant organisms!*
We apologize for the lack of clarity. In the paper by Bridgham et al., the authors combined functional characterization of sponge nuclear receptors (that are effectively extant organisms!) with phylogeny analysis and ancestral sequence reconstruction to show that "*NRs evolved from a ligand-activated ancestral receptor that existed near the base of the Metazoa, with fatty acids as possible ancestral ligands*".

To clarify we have modified the sentence page 18 as follows: "*Functional characterization of sponge receptors combined with phylogeny analysis and ancestral sequence reconstruction allowed Bridgham et al., to propose that NRs evolved from a ligand-activated ancestral receptor that existed near the base of the Metazoa, with fatty acids as possible ancestral ligands".*

8.*Page 19: "Thus, from the evolutionary point of view, the disappearance of π-turn in more evolved receptors facilitated the binding of a variety of molecules and promoted their diversification." "More evolved" in what sense?*

We apologize for the confusing sentence that we have modified as follows:

"*Thus, from the evolutionary point of view, the disappearance of the π-turn in more recent nuclear receptors (from NR1 and NR4 subfamilies) facilitated the binding of a variety of molecules and promoted their diversification*."

9.*Page 22: Why was the LG model with gamma law chosen for phylogenetic tree construction?*
The LG model is based on the most recent substitution matrix and therefore is most often the best for

protein alignments. The gamma law is a classical correction for parameter estimation using maximum likelihood. Following reviewer comment we performed extensive model testing using the AIC and BIC criteria as implemented in the SMS software (Lefort, V.; Longueville, J.-E. & Gascuel, O. SMS: Smart Model Selection in PhyML. *Molecular Biology and Evolution,* **2017***, 34*, 2422-2424). The model with the best likelihood was LG+G+I, so with additional estimation of the proportion of invariable sites. While this does not change significantly the topology of the tree and the branch support value, we updated the Supp Figure 3 according to the new model.

10.*Page 24: "....however when comparing the scores of both best models from the two modeling strategies, the model with π-turn has the best score (-27875.7) with respect to the one without a π-turn (-27771)...."*
*I think this interpretation is incorrect. The lower DOPE score relates to the fact that removing the closest homologs results in a less-accurate model.*

To further support the quality of the model with the π-turn observed in the structure, we used ProQ2 since it has the advantage to predict local and global quality of protein models based on sequence and structure features, such as atom-atom contacts and residue-residue contacts. Using ProQ2 to compute residue-based scores on either the model with and without π-turn, we calculated the average score of helix H7 (ranging from 0 to 1, the latter being the best score) and obtained 0.71 and 0.65 with standard deviations of 0.06 and 0.04 for each model respectively, supporting an enhancement in quality with the presence of the π-turn in the nuclear receptor structure.

11.*Page 26: ".....all the system was energy minimized with 2000 steps of CG and heating to 300K over 15ps, followed by 85ps of equilibration. The production run was then performed for the duration of 50ns*"

*The time scale used for equilibration is way too short - I wonder if the system really equilibrated. Was a simulation of only 50 ns enough to explore the conformational space of the complexes? I very much doubt it.*

The molecular dynamics simulation protocol has been carefully benchmarked across different nuclear receptor proteins throughout the last years and has been used on several systems, such as RAR, ER, GR for instance and published at numerous occasions (Bianchetti et al 2018, Chebaro et al 2017, Chebaro et al 2013).

Exploring the conformational space of the complexes is an intricate task to achieve and could require more extensive calculations. As the purpose of these simulations is to assess the stability of the modeled structures, we believe as per our experience with nuclear receptors, that 50ns is sufficient for the systems to equilibrate and allow us to hypothesize on the presence of the pi-turn, which we validate experimentally. In addition, the first 10ns of the simulations were extracted from the analysis to ensure a good convergence. We would like to further insist on the fact that these simulations were performed in order to estimate the structural stability of the pi-turn structure obtained in the homology model, and by no means to explore the conformational landscape of this subtle conformation.

We calculated the $C_\alpha$-RMSD of the three systems WT, ΔE352, and E352A as represented in the following figure in black, red and blue respectively, to further illustrate the stability of the structures.

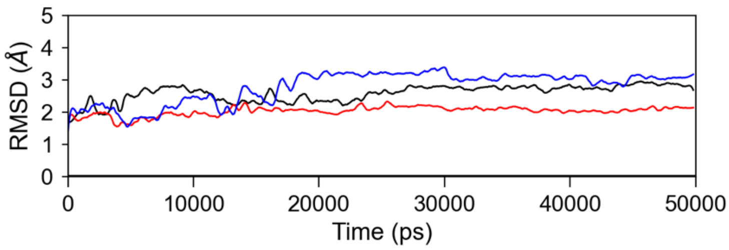


**Suppl. Fig. 6D**. Time evolution of the $C_{alpha}$-RMSD of the three systems wtRXR, ΔE352 RXR, and E352A RXR, represented in black, red and blue respectively, over the course of the 50ns molecular dynamics simulations.

*The values of the MM/PBSA analysis are presented without standard error, standard deviation, or any mention of autocorrelation times. It is, therefore, difficult to judge how meaningfully different they are.*

*Page 26: "Protein and solvent contribution to the electrostatics terms in the binding free energy were calculated using the University of Houston Brownian Dynamics (UHBD)...."*

*Details are missing here. What dielectric constants and salt concentration were used? Was a non-linear PB used?*

We thank the reviewer for pointing this out. The binding free energies were calculated on the average structures computed for each system between 10 and 50ns, which as presented above show stable time series, hence the absence of standard deviations or standard errors are provided as a qualitative comparison between the different models discussed in this work.

We used the UHBD program to solve the linearized Poisson-Boltzmann equation to compute the electrostatic binding free energy of binding of the two molecules. Van der Waals radii and charges of atoms are obtained from the force field CHARMM36. A dielectric constant of 80 was used for the solvent, 1 for the protein and a pH of 7. A non-bonded cutoff of 12.5 Å were used with a shift truncation function for electrostatics.

Although MM/PBSA does not take into account conformational entropy, our protocol has been validated on a variety of systems to assess protein/protein complexes in terms of relative free energies and proven to be in agreement with experimental data (Zanier et al Science 2013, Moroy G et al JBC 2009). Further details on the methodology were added in the materials and methods section.
